# Understanding hard-to-reach communities: local perspectives and experiences of trachoma control among the pastoralist Maasai in northern Tanzania

**DOI:** 10.1017/S0021932020000553

**Published:** 2020-09-25

**Authors:** Tara B. Mtuy, Kevin Bardosh, Jeremiah Ngondi, Upendo Mwingira, Janet Seeley, Matthew Burton, Shelley Lees

**Affiliations:** 1 Department of Global Health and Development, London School of Hygiene & Tropical Medicine, London, UK; 2 International Centre for Eye Health, London School of Hygiene & Tropical Medicine, London, UK; 3 Center for One Health Research, School of Public Health, University of Washington, USA; 4 RTI International, Washington, DC, USA; 5 NTD Control Programme, National Institute for Medical Research, Dar es Salaam, Tanzania

**Keywords:** Trachoma, MDA, Hard-to-reach communities

## Abstract

As progress to eliminate trachoma is made, addressing hard-to-reach communities becomes of greater significance. Areas in Tanzania, inhabited by the Maasai, remain endemic for trachoma. This study assessed the effectiveness of Mass Drug Administration (MDA) through an ethnographic study of trachoma amongst a Maasai community. The MDA experience in the context of the livelihoods of the Maasai in a changing political economy was explored using participant observation and household interviews. Factors influencing MDA effectiveness within five domains were analysed. 1) *Terrain of intervention*: Human movement hindered MDA, including seasonal migration, domestic chores, grazing and school. Encounters with wildlife were significant. 2) *Socio-cultural factors and community agency*: Norms around pregnancy led women to accept the drug but hide refusal to swallow the drug. Timing of Community Drug Distributor (CDD) visits conflicted with livestock grazing. Refusals occurred among the *ilmurrani* age group and older women. Mistrust significantly hindered uptake of drugs. 3) *Strategies and motivation of drug distributors*: Maa-speaking CDDs were critical to effective drug delivery. Maasai CDDs, whilst motivated, faced challenges of distances, encounters with wildlife and compensation. 4) *Socio-materiality of technology*: Decreases in side-effects over years have improved trust in the drug. Restrictions to swallowing drugs and/or water were relevant to post-partum women and the *ilmurrani*. 5) *History and health governance*: Whilst perceptions of the programme were positive, communities questioned government priorities for resources for hospitals, medicines, clean water and roads. They complained of a lack of information and involvement of community members in health care services. With elimination in sight, hard-to-reach communities are paramount as these are probably the last foci of infection. Effective delivery of MDA programmes in such communities requires a critical understanding of community experiences and responses that can inform tailored approaches to trachoma control. Application of a critical social science perspective should be embedded in planning and evaluation of all NTD programmes.

## Introduction

The term Neglected Tropical Disease (NTD) not only refers to the biological disease but alludes to an indication of the types of communities affected by these diseases of poverty. It implies the world’s poorest societies that are marginalized socially, politically and/or economically. Many communities burdened with NTDs are also hard-to-reach populations because of geography or socio-cultural difference (e.g. Bardosh, 2014a; Parker *et al*., 2016). The ‘Neglected’ in NTD refers to a disease’s status relative to HIV, TB and malaria – but also to prevalence among the world’s poorest and marginalized communities (Manderson *et al*., 2009). Emphasis is typically on the biological aspects of disease while neglect encompasses social constructs and livelihoods of a community (Parker *et al*., 2016).

The NTD trachoma (ocular infections with *Chlamydia trachomatis*) is the leading infectious cause of blindness. In 2019, WHO estimated that 142 million people live in trachoma-endemic areas globally (WHO, 2019). Whilst trachoma is estimated to affect 17% of the population of Tanzania (WHO, 2017), the majority of trachoma-endemic areas are predominantly inhabited by the Maasai – a pastoral ethnic group. The baseline prevalence of trachomatous inflammation–follicular (TF) in Longido District was greater than 50% in 2004 (Masesa *et al*., 2007; Mwingira *et al*., 2016). Longido District received more than five rounds of MDA before undertaking trachoma impact surveys in 2018, showing that TF had declined to 7.2%; however, MDA was continued as per the World Health Organization (WHO) guidelines. Prior to 2015, MDA coverage was patchy. Following concerted efforts to improve by the Tanzania NTD control programme and partners, coverage has improved gradually: 43% in 2015, 66% in 2016, 76% in 2017, 87% in 2018 and 94% in 2019.

Mass Drug Administration (MDA) of azithromycin is one of four components of the Surgery, Antibiotics, Facial cleanliness and Environmental improvement (SAFE) strategy for the elimination of trachoma. The other components are surgery for trichiasis, facial cleanliness and environmental improvement – including management of animal and human feces and access to water. MDA is coordinated by a number of international actors in partnership with government ministries in a vertical approach. Azithromycin (trade name *Zithromax*) is donated by the pharmaceutical company Pfizer to trachoma-endemic countries via the International Trachoma Initiative (ITI). In 2016 more than 85 million people received donated *Zithromax* for trachoma worldwide (WHO, 2017). The Tanzania national NTD control programme – along with implementing partners – oversees and coordinates these control measures. MDA campaigns are coordinated by the national NTD programme and implemented through a train-the-trainer model with local government offices at district and then village level. Distribution is done by Community Drug Distributors (CDDs).

While the prevalence of TF has been reduced in some hard-to-reach Tanzanian communities, evidence suggests that after three rounds of MDA in hyperendemic communities there has been a re-emergence of infection and at least seven rounds are required to attain a TF prevalence of <5% (West *et al*., 2011). These hard-to-reach communities are often marginalized communities that require a critical understanding of their social, political and economic context and therefore a tailored approach. Control of diseases in neglected communities often leads to tensions between local cultural demands and national targets. The same socio-political factors that drive NTD transmission in marginalized communities pose challenges in control efforts (Bardosh, 2014a).

There have been efforts to understand the context of MDA and the community response to NTDs, including local conflict, physical and social isolation, migrancy, side-effects, trust, rumours and disease knowledge (Babu & Kar, 2004; Burton *et al*., 2005; Desmond *et al*., 2005; Parker *et al*., 2008; Cavalli *et al*., 2010; Ssemanda *et al*., 2012; Hastings, 2013; Parker & Allen, 2013). In a vertical public health approach, often there is inattention to the socio-cultural, political and economic influences on the effectiveness of a programme. Reflections on the ways in which these issues influence MDA in hard-to-reach communities hyperendemic for trachoma are limited (Desmond *et al*., 2005).

To date there have been no studies detailing MDA for trachoma from a Maasai political economy perspective. The Maasai have complex livelihoods and are confronted with social and political challenges that may affect their interaction with such programmes. Although vaccination coverage is generally high, compared with other tribes, Maasai had lower reports of receiving vaccinations (ranging from 76 to 95%) and the overall health status of Maasai is poor compared with other Tanzanian ethnic groups (Lawson *et al*., 2014) implying a possible disconnect between the Maasai and health services. This study explored the Maasai experience of MDA using a socio-anthropological framework for assessing the effectiveness of NTD interventions, drawing on an ethnographic study of trachoma among Maasai in northern Tanzania. The aim of the study was to understand the response to MDA for trachoma against a backdrop of unique livelihoods and a changing political economy. Collinson (2003) defined political-economy as the ‘interaction of political and economic processes within a society; distribution of power and wealth and processes that create, sustain and transform these relationships over time’.

## Methods

### Study location

This study was based on ethnographic research carried out from September 2016 to December 2017, looking at Maasai’s experiences and perceptions of trachoma control in the Sinya Ward in Longido District in northern Tanzania, which is representative of other Maasai dwelling wards in northern Tanzania. Sinya is located in the plains between Mt Kilimanjaro and Mt Meru and is situated on the Tanzania–Kenya political border with Amboseli National Park in Kenya bordering Sinya on the north. This hard-to-reach community is 60 km from the nearest large town, Longido. There is no public transport to Longido and dangerous wildlife in the vicinity make the journey by foot hazardous. Sinya is situated within the Enduimet Wildlife Management Area (WMA) – a Tanzania government authority that manages wildlife resources and conservation outside the Tanzania national parks. Sinya is comprised of three villages – Il Donyo, Leremeta and Endonyoemali – with a total population of 4285. There are only two health dispensaries, one boarding primary school and one market held once per week to serve these three villages. Electricity was brought to the ward in 2016 but only serving the trading centre. In 2017 three boreholes were constructed but prior to then there was only one borehole in Sinya.

This community was purposely selected for this research for the following reasons: it was a trachoma-hyperendemic community; had a majority Maasai population; there was the cooperation of village leaders; and it was reasonably accessible to the lead researcher’s home town. Most of Sinya’s residents have permanent homesteads in the village. There are a few non-Maasai *ormeek* staying in Sinya for the purpose of government work in the schools and dispensaries and for trade. The main source of livelihood is traditional livestock production in this purely pastoralist community (Mtuy *et al*., 2019).

### Sampling

Sinya Ward has had five annual rounds of MDA of *Zithromax*, in 2015, 2016, 2017, 2018 and 2019. This study was conducted during the third round in 2017. It used qualitative methods, including participant observations and repeated household interviews (*n* = 40). The lead author had lived in this community for over 16 months, involving herself in the livelihoods of the community. Along with a Maasai research assistant, the lead author conducted participant observations in social settings such as the market, people’s homes, the community boreholes and celebrations; at two government dispensaries; during trichiasis surgical outreach visits; and informal discussions while accompanying Community Drug Distributors (CDDs) and village leaders during MDA in July 2017.

A ‘boma’, or *enkang* in Maa (Maasai language), is a homestead or joint residential unit composed of a number of households. The *enkang* allows for cooperative decision-making over pastoralist activities (Coast, 2001). Physically it is designed with a carrel in the centre for the cattle, surrounded by huts and all enclosed with a fence to protect the cattle from wildlife. A boma is headed by one male and in a polygamous culture, each wife with her children have a hut within the boma. Huts are built alternatively on the left and right side in order of marriage to wives. Married sons live in their father’s *enkang* until his death. The population of bomas vary but with a changing political economy the number of households within a boma is decreasing. In 1998 an *enkang* in Tanzania comprised of four households, compared with seven to nine in the 1960s (Coast, 2001). For the purpose of this study, a boma is considered a household with the male elder of the boma as the head of the household. Among the three villages of Sinya, there are a total of ten sub-villages and a total of 107 bomas. Random sampling of bomas was used to allow for transparency in the selection process within the community. The lead author aimed to reduce perceptions of favouritism in a society in which decision-making is based on fairness. Internet-based sample builder was used to randomly select two bomas from each sub-village (www.randomizer.org) for household interviews. If the first randomly selected boma was not available, the next boma in the randomization list was approached to participate. As the lead researcher and research assistant had been living among the community for 10 months at that time, most residents of the selected bomas were already familiar with the researchers and a rapport had been established.

Semi-structured household interviews were conducted in Maa by a native Maa-speaking interviewer in a conversation-like manner. The interview guide for the first round of interviews consisted of open-ended questions on their experiences and perceptions of health and non-health related programmes in the community. The second round of household interviews, conducted one-month post-MDA, consisted of open-ended questions on the MDA experience, perception of the programme, decision-making, migration and prevention of trachoma. Interviews were audio-recorded and later transcribed and translated from Maa to English.

### MDA procedures

Longido District had had five annual rounds of MDA for trachoma, in 2015, 2016, 2017, 2018 and 2019. Training, supervision and logistics were organized by the national NTD control programme with support from international partners. In a train-the-trainer model, the national NTD programme trained district-level trainers, who then trained ward-level trainers. In the case of Sinya Ward this was the Il Donyo dispensary doctor in charge. The ward-level trainer conducted training of Sinya CDDs the week prior to MDA. *Zithromax*, donated by the International Trachoma Initiative to the national NTD control programme, was delivered from the government to the district and then delivered to the doctor in charge at Il Donyo dispensary. Only medication for trachoma was distributed during this MDA. The MDA was conducted boma to boma by two CDDs per sub-village over the course of 4 days, although distribution was not done one of the days due to market day. A single dose of *Zithromax* tablet(s) dosed according to height was given to individuals above 7 years of age and taller than 120 cm according to national guidelines. Children aged 6 months to 7 years were given *Zithromax* paediatric oral suspension dosed according to height (International Trachoma Initiative, 2018). Each day of MDA, the lead author would accompany two groups of CDDs in a village. Ultimately, observations covered parts of MDA in all three study villages.

### Data management and analysis

Transcription of household interviews was done directly from Maa to English; some transcripts were corrected to ensure more understandable English while assuring meaning was not changed. English transcripts and field observations were entered into NVIVO 11 Software. Initial interpretation included familiarization of the data. Using a thematic content approach, data from interviews and field notes were first coded by the lead author, TM, and verified by author SL. Through data immersion, emerging themes were identified and confirmed against the study objectives.

**Figure 1:**
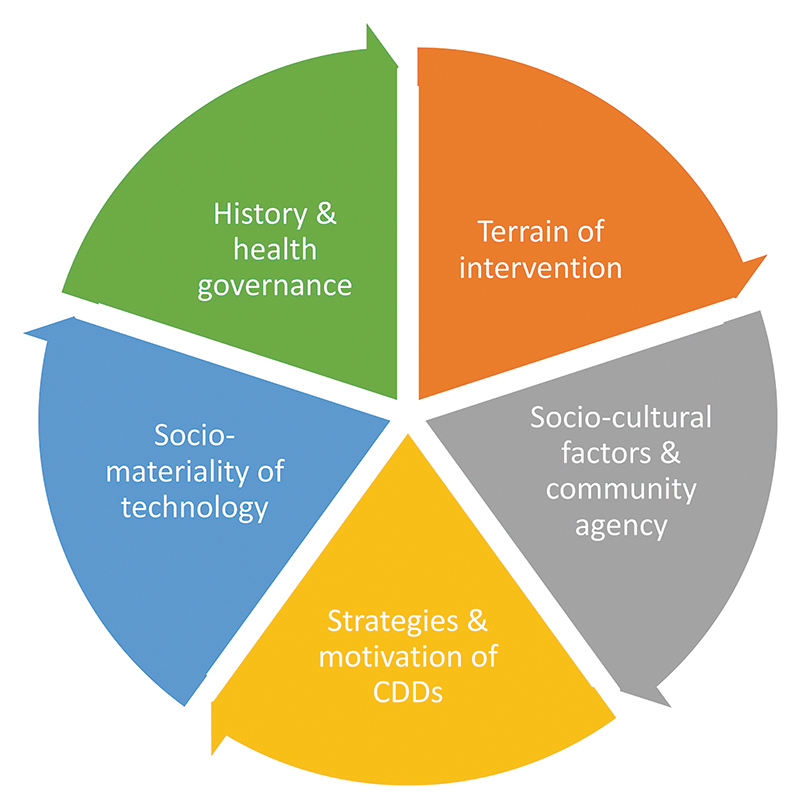
Five domains for assessing the effectiveness of MDA programmes for trachoma in Maasai communities.

Impressions and interpretation of the themes were discussed with the native-speaking interviewer and co-authors. Using an analytical framework adapted from Bardosh (2018), themes were classified into the framework domains (Figure 1). The domains were modified for the local Maasai context and for the type of data collected specific to trachoma. Narrative text was applied around the framework and direct quotes presented are used to show dominant views of participants.

## Results

### Domain 1: Terrain of intervention

Control programmes, specifically MDA, are delivered in short time frames in a space that, aside from socio-cultural practices and governance histories, is influenced by livelihood, climatic and geographical factors (Scoones, 2009). In Sinya Ward in northern Tanzania, seasonal fluctuations, human population movement and socioeconomic pressures all play an important role in influencing MDA delivery.

### Seasonal fluctuations and human population movement

**Figure 2:**
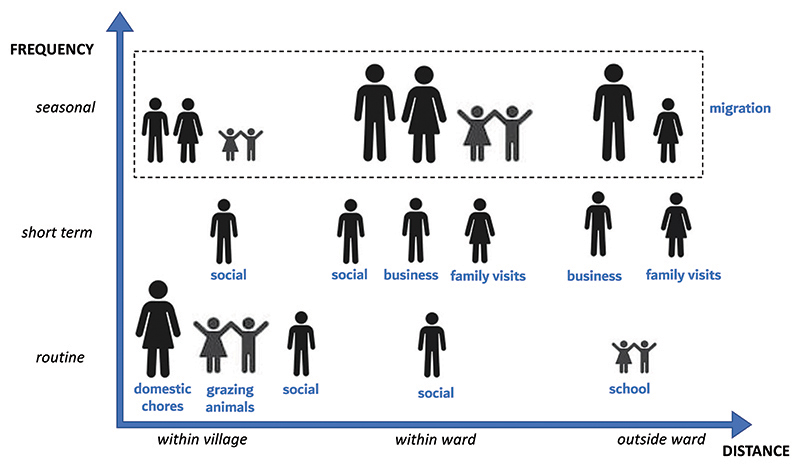
Human population movement during MDA, stratified by spatial (distance travelled) and temporal (frequency of travel) characteristics. Routine is within 24 hours. Short terms are greater than 24 hours and less than 2 weeks. Men, women and children are represented. The size of the symbol illustrates how common a particular human movement was during MDA.

Figure 2 shows human population movement during MDA in Sinya. The largest contributor towards movement was *seasonal migration*. Sinya is dependent on rainfall to maintain the pastoralist lifestyle of the local Maasai community. The MDA was conducted in July, typically a dry month but with green pastures after the long rains from April to June. In 2017, the rains were shorter than usual and it was considered a drought year. The village of Il Donyo, for example, has open spaces with green pastures that lie outside areas designated for permanent bomas. Therefore, some households migrated to temporary bomas (*ronjo*) to access these new green pastures, which were still within their village but administratively in another sub-village. This was typically done for the smaller livestock (goats and sheep) and involved the whole family. Temporary bomas are temporary housing structures outside designated community areas for permanent homesteads that are only used in times of drought. These are established by village leaders for land management purposes. Each of the three villages in this study had households who had migrated to temporary bomas within the ward but within different villages.

All CDDs observed during MDA were uncertain how to handle this migration. In most cases the CDDs assumed the CDD from their permanent sub-village would locate them and therefore they ignored them. For example, one of the larger bomas in Sinya Ward from Leremeta had migrated to Il Donyo village, approximately 18 km away. The CDDs from Leremeta said if the researcher (the lead author) was not present to drive them to the temporary boma they would not have covered this boma. On the other hand, some CDDs added those in *ronjo* to the registers of the new sub-village they were temporarily residing in, possibly falsely inflating the census for the village.

Yes I’m aware of that [MDA] however those CDDs didn’t give us drugs because those who came were distributing drugs for people from Il Donyo only and they told us that we have to wait for CDDs from Leremeta because every CDD has his/her area to cover. [boma 10-2]

We didn’t take them [Zithromax]. And not because we refused but because those CDDs didn’t come here to our temporary boma. [boma 10-2]

In almost all bomas visited during observation, most men had migrated outside the ward for up to several months in search for green pastures for larger cattle. This included the warrior group, *ilmurrani* (sing. *olmurrani*), aged approximately 15–30 years; some junior elders (*ilpayiani* or *ildasati*) aged approximately 30–50 years; and a few women. Children were always left behind in the ward with mothers and grandmothers, some to continue attending school and some to graze smaller livestock. In some cases, the men had migrated just outside the ward and the women reported that they delivered the drug to the men.

Ilmurrani were not at home [during MDA]. They migrated to temporary bomas with cattle. But because we do meet with them daily at the borehole we took their drugs to them. [boma 13-2]

Aside from seasonal migration, *routine human movement*, as shown in Figure 2, would occur within a day. Such movement affected the availability of community members when CDDs came to the bomas to distribute *Zithromax* over the four days of MDA. The largest groups of people moving on a daily basis were women attending to domestic chores, including walking long distances to fetch water and firewood; and children grazing smaller livestock. A few men would visit men of their age-sets either within the village or within the ward. In one case, after the exchange of cultural greetings between the CDDs and the elder of the boma, the elder excused himself to go visit a fellow *olpayiani* in the village. Due to age-grade differences between the CDD and the elder, the CDD was unable to question his decision to leave prior to being given the drug.

Many school-age children missed MDA. The village of Leremeta has many children who are day students at a school in Kenya. A few children were boarding outside of Sinya. The majority of primary school-aged children from all three villages were boarding at Sinya Primary School in Il donyo Village. Sinya Primary School has 928 students of which approximately 75% (*n* = 700) were from Sinya. Distribution was done for all children boarding at the school. For the purpose of documenting distribution to school children, the CDD explained that the school was listed as a ‘household’ rather than recording at the household level. Yet in some cases, children were listed in the census of the school as well as the household thereby inflating the total census.

Figure 2 shows *short-term human population movement* that occurred over a few days, greater than 24 hours and usually less than 2 weeks. This included men, usually *ilmurrani*, visiting other men in their age-sets but still within the village. Part of being an *ilmurrani* is moving around with fellow *ilmurrani* to engage in cultural activities together. Some men travel outside the ward for business, usually to neighbouring Kenya or Longido. Elder women (*koko*) would visit their daughters and grandchildren married out of the village either still within Sinya or outside the ward.

A grandmother from outside Sinya is visiting a boma in Sinya to attend a female initiation ceremony. She will stay for another two weeks. CDDs discussed her situation and decided that when she goes back to her home village, MDA will be finished and she won’t get the drug. So they decided to give her Zithromax and add her to the census. [field notes]

### Socioeconomic pressures

A related issue involves the abundance and seasonal distribution of wildlife in Sinya Ward, which is also a challenge to the livelihoods of the local Maasai pastoralists. During MDA in July 2017, there were two separate hyena attacks on cattle. In one case, 36 goats and sheep were attacked by hyenas at night. When the CDD arrived at the boma, men were out searching for the remaining cattle and women and children were upset and distracted by the incident and did not want to receive or to use the *Zithromax*.

### Geographic terrain

The geographic terrain also inhibits the movement of CDDs. For example, elephants were passing through the village of Endonyoemali during the week of MDA, preventing CDDs from moving from boma to boma to distribute *Zithromax*. The CDDs also reported covering long distances as sub-villages are spread out over a large geographical area. Thus, such distances are not easy to cover by foot in regards to time and dangers of encountering wildlife and bicycles are not available in Sinya.

### Domain 2: Socio-cultural factors and community agency

The Maasai have a very strong sense of cultural identity, which is expressed in beliefs, norms and traditions. There is a tendency for public health interventions to expect communities to place high importance on their programme with poor consideration of how interventions fit into the communities’ priorities and the complexity of livelihoods (Parker *et al*., 2008; Bardosh, 2014b; Hastings, 2016; Martineau *et al*., 2017). Communities are a social network influencing norms and behaviours. Control programmes need to understand and account for socio-cultural influences on individual and community decisions specific to marginalized communities in particular.

### Disclosure of pregnancy

Maasai tend to have high fertility rates due to a desire for large families. However, due to social convention women do not publicly discuss their pregnancy status, especially with men. This poses a challenge to CDDs, most of whom are men, as *Zithromax* is not to be dispensed to pregnant women. Despite CDDs explaining this at each boma, no women observed disclosed that they were pregnant. Maasai wear a traditional cloth, the shuka, and typically, women wear a few layers of shukas loosely wrapped around their waist and on their shoulders. Due to the multiple layered clothes pregnancy is easily concealed. Instead of disclosing, women tended to imply their pregnancy through refusal.

**Table:** 

Woman 1	I can’t take it.
CDD	Why?
Woman 1	I have a small problem.
Woman 2	I can’t take that. I may vomit.

In response, some CDDs would ask if they had problems with their stomach – an indirect and acceptable way of asking if they were pregnant. Not all pregnant women took this action, but rather concealed the drug, being likely to dispose of it later.

One mama was too shy to say she’s pregnant so she accepted the drug and pretended to take it but didn’t. She knew we saw so she started laughing along with all the other mamas. [field notes]

During one household interview women explained that the CDD left drugs for those not present but these were not consumed by the returning pregnant women. In such cases, CDDs recorded these as administrated drugs, which falsely inflated coverage. While observing MDA at one boma, CDDs estimated there were eight pregnant women in that boma, without evidence. As there was no direct confirmation of pregnancy or reporting, it was impossible to know if these were refusals for other reasons.

### Social norms

In a pastoralist Maasai community, tending to the cattle is top priority, which was observed when visiting bomas. Traditionally livestock are viewed as a store of wealth, a source of food and symbol of prestige (Hodgson, 1999). Bomas are busy in the mornings, requiring participation from most of the household, including rounding up cattle, checking for illness, milking, spraying or washing cattle with insecticides, and making plans for grazing with young herders. While nearly all respondents reported this time (around 7 am) as the ideal time to find most people home in the boma, tending to the cattle was their priority. While women and children may have been available, the established norm is to first sit down with men, in order to ‘share the news’ (*anya ilomon*) and seek permission from the male head of the boma and other men to administer the drug. However, during early morning visits with CDDs men were often busy attending to cattle and CDDs were given limited attention and a low priority.

### Decision-making/social relations

In the socially tight-knit community of the Maasai, individuals can heavily influence the views and decisions of others. Some are more cooperative than others and some refuse, influencing the choices of their family members and neighbours.

Although women said that decision-making around uptake of *Zithromax* was theirs to make and permission from men was not needed, decisions were influenced by their female peers. There were some cases of active refusal from mothers and grandmothers who walked away with harsh words about the drugs or the programme.

Elder woman: If you don’t come with clean water to distribute drug then we don’t take it.

Elder man: Why you causing problems? You use this dirty water everyday. Why is it a problem today? Stop it! [field notes]

In another case an elder woman said ‘I’m leaving because I have nothing to do with this programme’. The CDD explained the drug was for everyone, including older people. The elder woman said ‘I’ll come back later’. She never came back while the CDDs were at the boma.

In some of these instances, those that ‘accepted’ did so with hesitation and were not seen swallowing the drug. It was thought some of these cases may have disposed of the drug when CDDs left. The *ilmurrani* age group were observed walking away from the boma when CDDs arrived or refusing *Zithromax*. This is probably due to norms of collective decision-making among this male group. In Maasai culture, groups are based on age and rites of passage, which are an important means of defining your place in society and norms that are to be adhered to. *Ilmurrani* are circumcised men, considered warriors. Traditionally they do not marry but are expected to be sexually active. Historically their role is for protection of livestock and property and therefore are exempt from herding. Although *manyattas* (warrior camps) are less common, *ilmurrani* still spend most of their time with fellows forming strong bonds through various cultural traditions.

They [ilmurrani] didn’t take [drug] because they were not around at the boma. So I’m unsure if they were around they would take or refuse. Also ilmurrani do not easily swallow anything. [boma 20-2]

Some ilmurrani refused the drug. Yes my husband [olmurrani] refused to take the drug. [boma 09-2]

The influence of leaders and the people’s relationships with CDDs motivated some community members to participate in MDA. Two of the three villages were well informed of plans of MDA and its benefits by village and sub-village leaders. This was evident from the level of cooperation among these residents compared with the one village having less-active leaders. Here the residents complained they had little information about the programme and showed uncertainty or asked a lot of questions.

Studies have shown higher coverage when CDDs are living close or within the communities (Emukah *et al*., 2008; Katabarwa *et al*., 2010; Parker & Allen, 2013). In this study people respected and trusted some of the CDDs that they knew and were comfortable with, which motivated them to take *Zithromax*. Consistency of CDDs positively impacted coverage; one CDD observed during the 2017 MDA had worked in the same sub-village for all 3 years of *Zithromax* distribution, which had clearly influenced the high level of trust and positive response to the MDA.

### Rumours

There were some misconceptions among women that Zithromax was for family planning. Although when asked at the household interviews, all denied this belief and provided evidence to say it wasn’t plausible.

That [Zithromax is for family planning] is not true because we took these drugs for three years now but women continue to carry. [boma 04-2]

Yet some reported they heard this rumour but not at their boma,

That [there is a rumour that MDA is for family planning] is true but this boma has no beliefs like that. [boma 20-2]

Others linked trust or mistrust in MDA with experiences with trichiasis surgery. One boma (01-2) explained that because many women had no improvement after trichiasis surgery, and this discouraged them from participating in MDA.

There was a woman who had trichiasis surgery at this boma and now she is ok and that is why we trust you and your drugs now. [boma 11-2]

### Local understandings of disease

Local biomedical understandings of disease in general, of disease prevention and, more specifically, of trachoma were relatively poor in Sinya Ward. The Maa term *enaoji* given for eyelid irritation is not specific to trachoma but is recognized as a problem among Maasai (Mtuy *et al*., 2019). *Enaoji* is associated with environmental conditions or linked with supernatural influences. Prevention was not well understood and MDA was considered only for the purpose of treatment (Mtuy *et al*., 2019). The value placed on the control programme was low, in line with this local knowledge and this affected people’s motivation to participate. Health education by CDDs was also observed to be brief and inadequate.

On the first day of MDA in 2017, CDDs offered biomedical explanations for trachoma and the benefit of *Zithromax* but explanations became notably shorter on subsequent days of MDA. In most cases, the CDDs made people feel comfortable to ask questions. Although overall there were few, most questions asked were related to clarification on side-effects. Further explanations did not incorporate local understandings or benefits specific to the community at large. Specifically, the reason for ‘mass’ distribution (versus only for those who were ‘sick’) and the importance of a majority of the community participating was never questioned nor explanations provided by CDDs, probably because CDD training did not cover this information.

There was confusion among the women distinguishing MDA from vaccination. They say the vaccination programme came to the area a few weeks ago for the kids. The CDD explains this is different and is for all people [little explanation on trachoma and the importance of the programme]. [field notes]

#### Domain 3: Strategies and motivation of CCDs

Despite the national NTD control programme providing adequate training materials and train-the-trainer programmes for MDA, there were adaptations to the delivery of the programme. This may have been done purposely to accommodate the local community and their needs or *ad hoc* as they were uncertain how to interpret guidelines for localized circumstances. The CDD training was conducted for the three villages together at Sinya Primary School.

### Selection of CDDs

Selection of CDDs was challenging due to local interpretation of requirements that they can read and write, had completed primary school and were 35 years of age or older; few people in Sinya met these criteria. This was a misinterpretation of national guidelines that they were only to be able to read and write and come from the same community. In most cases CDDs were Maasai community members (*n* = 15) but as some residents of sub-villages did not meet the CDD criteria, four non-Maasai working in Sinya were selected as CDDs. A non-Maasai government worker was a CDD, for example. This person was not respected by the community, who claimed he spoke harshly to community members and was not trusted. One resident said ‘He goes to MDA training to get allowance but did nothing with MDA… They are not serious.’ This CDD avoided going boma to boma and requested that community members report to the dispensary for MDA. Many did not attend, either because of their poor relationship with this CDD or because of the distance they needed to walk to the dispensary and the danger of encountering wildlife.

Maa is the primary language of Sinya and many residents did not speak nor understand Kiswahili. The non-Maasai CDDs (21%) were unable to speak Maa and this proved challenging when trying to provide information on MDA and trachoma, and in one case to even pronounce and verify names on the registers. People responded more positively to those CDDs they were familiar with and who spoke Maa. Some remarked more negatively about non-Maasai CDDs, the Swahili boy or the Mchagga (person of the Chagga ethnic group). At one boma, the non-Maasai CDD read aloud the name of the male head of the boma. Very upset, an elder woman said, ‘why are you calling his name? He is Papa. It is disrespectful to call an elder by his name.’

### Compensation

Overall the Maasai CDDs were extremely motivated to support the programme – in their words ‘to contribute toward improving the health of their people’. In many cases they walked 8 km in a day, spent extensive time at bomas to comply with Maa greetings and norms and in some cases used their own money to hire transport to reach bomas further away or at risk of wildlife attacks such as from herds of elephants, lions and cheetahs. Despite the national programme following WHO guidelines for CDDs, which define the role of CDDs as volunteers (WHO, 2020), the community perceived it differently. Maasai CDDs reported low compensation, lack of assistance with transport and inconsistencies in CDD training over the 3 years of MDA, including poor quality of training, delays in compensation and unfair selection of trainees. Similar complaints of lack of financial incentives, distances and inadequate supervision among CDDs have been seen in numerous studies (Emukah *et al*., 2008; Katabarwa *et al*., 2010; Parker *et al*., 2012). At a ward meeting for the introduction of this research in September 2016, many freely spoke about their perceptions of MDA. A few spoke about the demands placed on CDDs. They talked about the challenges faced by these volunteers, including locating migrating residents, distances travelled, encounters with wild-life and poor compensation. They also talked about the unfairness of inconsistencies of compensation to CDDs among villages within the same district. Informal conversations with CDDs indicated mistrust of the district’s management of MDA. The CDDS reported that in the second year of MDA (2016), only six out of twenty CDDs were selected to go for training, leaving many uninformed on MDA procedures. In 2016, while these figures were not verified, from the perspective of participants in this study, the payment to CDDs after distribution was 42% less than in 2015. In another village within the same district, CDDs demanded payment greater than a 3-fold increase and the district agreed. The CDDs questioned if compensation was provided as per the programme budget if such an increase for one village was allowable. In 2017, despite one district-level worker’s support and enthusiasm to accompany the researchers during MDA, she refused when allowances did not meet her expectations.

### Transport

Due to geographic distances and time constraints, most CDDs did not re-visit bomas to re-attempt to deliver MDA to those absent during their initial visit. The delivery of MDA, therefore, in this widely dispersed community, with neighbouring bomas being up to 2 km apart, was a one-off visit. In cases of residents who were not at the boma during MDA but were still located within the ward (either ‘routine’ or ‘short-term’ population movement), CDDs estimated the height of individuals with the assistance of family members and left behind the appropriate number of *Zithromax* tablets with the women for family members to take on their return. In such cases, swallowing of tablets, pregnancy status or updates to the census were not known nor accurately documented. This potentially led to overestimating coverage.

#### Domain 4: Socio-materiality of technology

Bardosh (2018) described NTD health interventions – in this case MDA – as a technology that is embedded within social relationships. *Zithromax* as a technology is perceived differently in different social contexts and by different social groups. In particular, side-effects, restrictions of drinking water for post-partum women and *ilmurrani* posed challenges for the community to accept the drug despite any understanding of the value of MDA for control of trachoma. *Zithromax* was often referred to as *indunda naado* (that red pill), or described as ‘big’ and ‘pills they had to take many of’. One participant (01-1) spoke of ‘the pill with corners’. Although Western medicine is accepted and used by residents of Sinya, physical appearance and possibly the meaning they attribute to *Zithromax* was different compared with other biomedicine.

### Side-effects

*Zithromax* was distributed for 3 years in Sinya and people either experienced first-hand or heard of others who had side-effects including vomiting, diarrhoea and, less commonly, headaches and dizziness. In one instance, a girl about age six was observed vomiting just minutes after taking *Zithromax*. Most people reported that the number of people complaining of side-effects were less each subsequent year. They mentioned that height sticks were not used in the first year and this may have contributed to the large number of reported side-effects. Some were not bothered by the vomiting and diarrhoea since that is a common local Maasai treatment for many illnesses – a form of cleansing and ridding the body of illness (Sindiga, 1995).

### Restrictions due to norms

Post-partum women are restricted to drinking only tea for the first 6 months post-delivery. Some post-partum women refused to take the drug because they could not take it with water and the option of taking it with tea was not raised. Others did so after other women told them it was OK for the purpose of taking medicine. Others had requested clean water in order to swallow *Zithromax*. Only one CDD carried a bucket of water for taking with the drug, although it was not clean. One of the restrictions for the *ilmurrani* age group (men aged approximately 15–30) is that they are not allowed to consume any food, beverage or medicines alone. This can only be done in the presence of a fellow *olmurrani*. During MDA, there were few *ilmurrani* in Sinya due to migration and therefore some *ilmurrani* either refused or put the *Zithromax* away until they met with a fellow *olmurrani*, and it was uncertain if they took the drugs or not. In these circumstances CDDs still recorded individuals as taking *Zithromax*, potentially inflating coverage figures.

#### Domain 5: History and health governance

The Maasai of Tanzania have a history of socio-political subjugation stemming from land allocation and lack of inclusion in the colonial government (Wagner-Glenn, 1992; Hodgson, 1999, 2001). According to Hodgson (2011), the lack of health facilities and schools in their communities, poor health services in urban settings due to language barriers and the different views on their development with that of the government and international donors have resulted in their demands for basic human rights. More recently, Maasai were evicted from part of the Serengeti National Park to create a hunting reserve for the Dubai royal family (Smith, 2014). Access to schools, health facilities, water and roads is still limited compared with the rest of the country (Sikar & Hodgson, 2006). For example, the Maasai have low vaccination coverage, which has been attributed to poor engagement with, and availability of, health services in their areas (Lawson *et al*., 2014). The political-economic context also had a major influence on responses to MDA, most visibly in the community’s lack of trust in the governments approaches to delivery of programmes and responses to community concerns including vertical approach of allocating public health resources and misalignment of priorities requiring assistance, namely water and animal–human conflicts.

#### Mistrust

A lack of trust in non-Maasai (*ormeek*) visitors was evident in Sinya. Many *ilmurrani* would run into the bush at the sight of the researcher’s Land Rover, which is similar to many government vehicles. The elders would laugh and remark that they thought the researchers (when driving CDDs that were being observed) were the government recruiting them for the military. Girls who did not attend school would hide when the researchers arrived at their homes in fear that they would be brought to school as per government regulations but against their fathers’ will.

Many did not know who was involved in the MDA programme beyond CDDs and the village doctors. Although MDA was supported by the national NTD control programme and international partners, participants were concerned that representatives did not visit Sinya around the time of MDA. A few attributed the programme to *wazungu* (white people) or ‘a white woman’, suggesting either the lead author or a woman who previously provided deworming drugs. People complained of lack of information and involvement of community members in the MDA programme. When the responsible government programme and international partners involved in trachoma control were explained, people showed more respect for the programme.

The drugs come from mzungus [foreigners]? (in a positive tone) [boma 11-2]

Government should tell us [about partners] and appreciate everyone’s contributions. [boma 14-2]

#### Misalignment of community versus government’s priorities

Whilst perceptions of the programme were positive, the community questioned government priorities, including lack of resources and access to hospitals, medicines, clean water and roads. Many said the MDA programme was beneficial to the community but asked why the government couldn’t provide access to adequate health services, clean water and roads. People were sceptical about the government’s efforts to control trachoma while the community’s concerns and needs for government assistance in other areas were ignored. A vertical approach to the distribution of public health resources from district to front-line primary health facilities and programmes had proved ineffective. Availability of drugs in the dispensaries was limited and treatment restricted due to a lack of specialized medical skills. For some residents of Leremeta, it was a 17 km journey to the nearest dispensary in Il Donyo. The closest hospital was 60 km away in Longido. There was no public transport to Longido and the journey by foot dangerous due to wildlife. During the time of this study, the only health programmes visiting Sinya were the district vaccination programme, trichiasis surgery camps and Marie Stopes for family planning services. While a number of NGOs that provide health programmes to Maasai communities were interviewed, most didn’t work in Sinya, stating it was too remote.

We would like if they use the resources [for MDA] to build a hospital which will have drugs for trachoma and other diseases. [boma 10-2]

We would like if they [the government] bring a hospital near to us because wild animals are a big challenge to us, especially elephants. So we wish they [the government] will help to solve this challenge because we travel many kilometres to go look for health services. [boma13-2]

Water is scarce in Sinya with only three boreholes for the ward. Two are powered by a generator, so only in operation a few hours a day, and one a manual pump. Within 3 months following MDA, the government improved the existing boreholes using solar power. Some thought this was part of the MDA programme and were appreciative.

I think the programme is OK because for the first time we have clean water by which the government put in boreholes. [boma 11-2]

#### Community-specific concerns not addressed

Animal–human conflict is a common occurrence in Sinya – something of major political interest. People remarked on the lack of government involvement or compensation for livestock killed by wildlife while their neighbours on the Kenyan side are compensated by the Kenya government. This issue was raised by residents of Sinya in everyday discussions and during household interviews when asked about perceptions of programmes working in Maasai communities. Two NGOs focusing on the protection of wildlife and reducing human–animal conflict previously worked in and around Sinya. Reasons for why the organizations left Sinya were unclear. Some explained that the NGO saw the community as uncooperative and not wanting to let go of their traditions of hunting lions among the *ilmurrani* age group. Others said they did not hunt for traditional reasons but rather killed wildlife in retaliation for attacking their cattle. They claimed the government doesn’t provide assistance to reduce the conflict or compensate people for losses from wildlife.

The community’s perceptions that the government has failed to fulfil promises has potentially impacted their trust in government programmes and officials. In a community meeting with the regional commissioner in November 2016, Sinya residents complained about not receiving promised compensation from a foreign aid project giving to those living on less than US$1 per day and compensation from the 2009 draught promised to pastoralists by the previous president. Historically, similar situations have been documented related to government promises to the Maasai on land use (Hodgson, 1999). Many complained of tensions created from tourist camps in that the land rent paid via Wildlife Management Authority hasn’t been seen by the community. Despite potential local government interference in how money is used, the community blamed development actors (such as NGOs) for these disputes.

## Discussion

**Figure 3:**
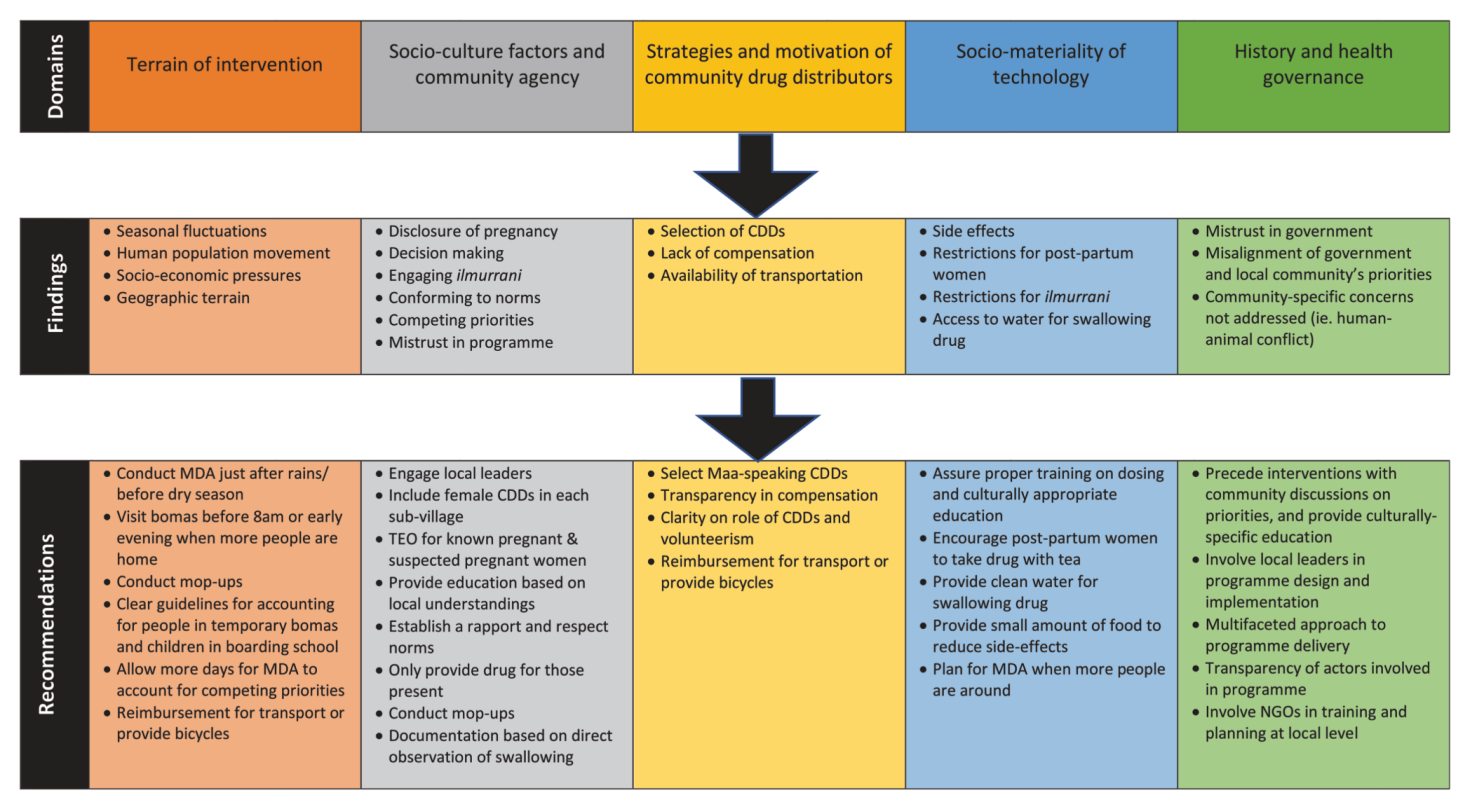
Findings and recommendations for planning control programmes.

This study identified factors specific to Maasai communities that contribute towards the effectiveness of delivering a mass distribution programme of *Zithromax* for trachoma. This research uncovered social, economic and environmental barriers to programme implementation, but furthermore the effects of a complex political economy. The implications of these findings and planning recommendations are detailed here, and highlighted in Figure 3, according to five domains.

Climatic conditions are central to the livelihoods of pastoralists and impact the optimal timing for community-based interventions. Similar seasonal challenges were seen in a rabies control programme among pastoralists in Tanzania (Bardosh *et al*., 2014) and interventions competing with the harvest season (Bardosh *et al*., 2014; Bardosh, 2015). Mass distribution administration should be planned around the seasons to reduce the frequency and distance of human population movement and reduce the stress that comes with draught and missed economic opportunities. Accounting for the complexities of human population movement in this Maasai community, the programme should consider additional resources to allow for return visits and travelling to those who are temporarily relocated outside their village. This would improve actual coverage and not rely on assumptions that individuals would take the drug left behind with family members. It is likely coverage was falsely inflated based on this assumption. Although rates of trachoma have decreased in these communities, research has shown in the absence of socioeconomic changes, the prevalence of NTDs often return to former levels (Nikolay *et al*., 2015; Amin & Abubaker, 2017). The high volume of human population movement among Maasai communities needs to be considered in regards to the reoccurrence of infection, as seen in some migrant trachoma-endemic communities (Burton *et al*., 2005; West *et al*., 2015). Compliance, which should be differentiated from coverage, is the frequency in which individuals have taken the drugs over repeated treatment rounds (Bockarie *et al*., 2013). Special attention needs to be given to non-compliers as potential sources of reinfection.

Although this community recognized trachoma as a problem (Mtuy *et al*., 2019) it was not a priority against a backdrop of complex livelihoods. Other ‘needs’ took precedence making it clear that MDA should work around those needs. This is not unique to the Maasai or to trachoma control but seen more broadly in countries of poverty (Parker *et al*., 2012; Bardosh *et al*., 2014; Bardosh, 2016). The push for programmes to demonstrate high coverage and value for money obscure the need to articulate social realities in the communities. Additionally, rumours and refusals were evident in the community, partly owing to misinformation. Rumours may also have historical or political origins or result from cultural beliefs (Parker *et al*., 2008 Hastings, 2016). Rumours of health interventions causing sterility or containing contraceptives have been documented since the 1920s in Africa, including vaccinations, malaria treatment and vitamin supplementation. Rumours of drug-induced sterility, as well as causing cancer, were reported in MDA for trachoma in Tanzania (Desmond *et al*., 2005). The act of passing on rumours may not be related to whether the person passing it on believes the rumour (Geissler & Pool, 2006) but rather a response to someone’s mistrust or uncertainty in something. It is critical that rumours be recognized, analysed and addressed, rather than seeing them as impediments to a public health programme (Kaler, 2009).

Knowledge of trachoma is poor in this community (Mtuy *et al*., 2019) and the education provided by CDDs was observed to be being minimal. A common belief is that if one is feeling well then there is no need for treatment (Desmond *et al*., 2005; Parker *et al*., 2008; Ssemanda *et al*., 2012). In another study in Tanzania (Desmond *et al*., 2005), sensitization was also poor, with 56% of respondents being satisfied with the amount of information they received prior to *Zithromax* distribution. Additionally, poor knowledge transfer can be linked to inadequate financial incentives for CDDs and village leaders (Bardosh *et al*., 2014).

Additional sensitization should be done with the *ilmurrani* age-set including their leaders and elders. Local leaders can help mobilize this more resistant group and engage them in meetings and education. There is motivation to help within the community when fair and transparent compensation is promised. Strategies for distribution among this group can be done more collectively to avoid cultural barriers such as issues of masculinity and conforming to norms and peer choices. Importantly, such strategies require financial incentives.

All people ineligible for *Zithromax* should get Tetracycline Eye Ointment (TEO) as per ITI guidelines. Guidelines in Tanzania should be updated to include TEO for known pregnant and suspected pregnant women. The MDA programmes should consider including a female CDD in each sub-village to engage with women. Female CDDs can provide more education and solicit more information from women to assure dispensing of appropriate forms of antibiotics and improve actual coverage. With high fertility rates in Maasai communities, there is a potential reservoir of untreated *Chlamydia trachomatis*. A combined programme of MDA with F (facial cleanliness) and E (environmental) components of SAFE may reduce infection, as seen in Sudan (Ngondi *et al*., 2006) – particularly as transmission is highest between mothers and children (Congdon *et al*., 1993).

Despite self-motivation of Maasai CDDs, the short-falls of the programme had a trickle-down effect. The CDDs were straddling the context of the programme and the interests of their community. Limited resources, pressures for high coverage and challenges with actors embedded in the programme left CDDs frustrated and less motivated to provide much needed education during MDA. Similar to other control programmes, long distances to be covered (Parker *et al*., 2012; Parker & Allen, 2013; Bardosh, 2016) and possible encounters with wildlife were the main barriers to CDDs delivering MDA to the community. Programmes could consider resources for transport, either through reimbursement or providing bicycles. Furthermore, the national programme’s expectation of the role of CDDs and their notion of volunteerism should be communicated prior to MDA and be consistent across the district to avoid misunderstandings. Additional catchment areas assigned to CDDs should be well estimated to assure CDDs can carry out responsibilities effectively – providing education, covering long distances, conducting mop-ups and engaging leaders.

Side-effects of *Zithromax* were commonly reported, as seen in other studies (Desmond *et al*., 2005; Astale *et al*., 2019). This is likely to be partly due to improper dosing. In some instances, CDDs didn’t use the dose poles and estimated doses. Additionally, side-effects are probably due to having empty stomachs when swallowing the drug. This should be considered in timing of distribution – or better still, programmes consider resources for providing a small amount of food and water during distribution.

This control programme is situated within a context of politics, history and a shifting economy, possibly influencing the response to the government and donor-led programmes. Land tenure policies created by the colonial administration in the early 1900s, many of which still exist today, were an effort to consolidate or isolate the Maasai and their cattle into designated areas and restrict their movement and interactions outside those areas (Wagner-Glenn, 1992; Hodgson, 1999). Examples of resistance are noted in their history, including the reaction to the colonial administration’s veterinary policies (Hodgson, 1999) and poor guidance by international NGOs focusing on the indigenous movement for Maasai (Hodgson, 2011). More recently, media reports have indicated a continued sense of political subjugation, marginalization and cultural discrimination of the Maasai (Magubira, 2014; Mjema, 2014; Smith, 2014). The traditional lifestyle of the Maasai is fragile, with changes in traditional migration due to reduced access to land and changes in weather; shifts in gender roles as women demand more ownership rights and decision-making power, and economic shifts due to drought and reduced grazing land, lead men to migrate for work and women to seek income-generating projects. This historical context and the cultural shift have possibly led Maasai to be uncertain and suspicious of government programmes. Although appreciation was expressed for the government’s efforts with this programme they questioned the lack of transparency of local and international actors involved. Repeatedly they felt frustrated by what they perceived as conflicting messages – distribution of drugs for NTDs yet essential needs for water or dispensaries with available medicines are not met. Other studies in Tanzania have seen similar barriers related to the nature of vertical programmes and the involvement of NGOs, government and local communities in the control of NTDs (Madon *et al*., 2014; Mubyazi *et al*., 2004; Parker *et al*., 2008; Samsky, 2011; Bardosh *et al*., 2014).

This study had some limitations. The lead researcher, a non-Maasai or *ormeek*, was aware of potential misunderstandings of her intentions of residing in the community. She may have been seen as representing NGOs or the government associated with MDA. To assure understanding of her position, the lead author lived in Sinya for 10 months prior to MDA and spent a significant amount of time engaging community members and local leaders in the research. It is also possible that the lead author’s presence during MDA could have altered the choices and behaviours of the community. Her presence may have motivated trainers to conduct more thorough CDD training, CDDs may have provided more education during distribution and community members may have been less resistant to the drug in the presence of a trusted outsider. The lead author assisted in providing transport to CDDs and community members in a few instances. Although this was only done in cases in which households had temporarily moved out of the sub-village, it is possible that it could lead to future expectations that the programme support transport for CDDs. With coming face-to-face with the realities of communities, field researchers are often in a position to empathise with the needs of those in the community (Kingori, 2013). This study did not verify reports of programme planning and implementation from the national programme and at district level as the focus was on the perspective of the community. Further to this, the representativeness of Sinya to other Maasai communities in Tanzania remains unclear. This purely pastoralist community may over-represent human population movement compared with agro-pastoralist Maasai communities, for example. Despite this, it is important to note that the communal nature of Maasai culture is still very traditional and consistent across different Maasai districts and economic levels.

In conclusion, implementing MDA programmes at the district level could be more effective as a joint SAFE programme rather than implementing them independently. To assure more accurate documentation of coverage, CDDs should distribute drugs only to those present as per guidelines, using a Directly Observed Treatment (DOT) approach. Due to a high degree of human population movement in this population, return visits and tracking of migrating individuals would assure improved coverage. Use of electronic data capture would allow tracking of individuals and the distribution of drugs to those who may have temporarily migrated from another sub-village. Education underpins all five domains. Knowledge and transparency allow the community to make more informed choices, avoid misunderstandings and rumours and improve respect for control programmes, ultimately improving cooperation and uptake.

As the end-game of trachoma elimination is near, hard-to-reach communities are likely to be the last foci of infection. Effective delivery of MDA programmes in hard-to-reach communities, including Maasai, requires a critical understanding of community experiences and responses that can inform tailored approaches to trachoma control. It comes down to a commitment of additional resources as the current strategies will not achieve similar outcomes in these hard-to-reach communities of poverty. Application of a critical bio-social perspective should be embedded in planning and evaluation of all NTD programmes.
